# Color Doppler Ultrasonographic Examination of Ovarian Grafts in Goats

**DOI:** 10.3390/vetsci11110580

**Published:** 2024-11-19

**Authors:** Antonio Renilson Sousa Vieira, Francisco Carlos de Sousa, Celso Henrique Souza Costa Barros, Maria Janiele Santana, Benner Geraldo Alves, Dárcio Ítalo Alves Teixeira

**Affiliations:** 1 Laboratory of Diagnostic Imaging Applied to Animal Reproduction, State University of Ceará—UECE, Av. Silas Munguba, 1700, Campus Itaperi, Fortaleza 60714-903, CE, Brazil; 2 INTA University Center—UNINTA, Rua Lucimar, 637, Dom Expedito, Campus Sobral, Sobral 62050-140, CE, Brazil; 3 Federal Institute of Education, Science and Technology of Ceará—IFCE, R. Carlos Antônio Sáles, s/n—Floresta, Campus Umirim, Limoeiro do Norte 62660-000, CE, Brazil; 4 Conception Biosciences, Fifth St, 2246, Berkeley, CA 94710, USA

**Keywords:** ultrasound, ovarian transplant, blood flow

## Abstract

The aim of this study was to evaluate the effectiveness of color Doppler ultrasonography for examination of the blood flow areas in superficial grafts after 7 or 15 days of heterotopic allotransplantation, comparing an in-ear subcutaneous area (IE) with an in-neck cervical intramuscular area (IN) in goats. To monitor the local blood flow in the graft areas, color Doppler signals were assessed daily until days seven and fifteen, when the left and right implants, respectively, were removed. The areas of blood flow around the transplanted ovarian fragments were significantly larger (*p* < 0.05) in the IE area compared to the IN area after 7 (IE: 4.70 ± 0.33^A^ vs. IN: 3.67 ± 0.33^B^) and 15 (IE: 5.27 ± 0.21^A^ vs. IN: 4.66 ± 0.22^B^) days of transplantation. A positive and significant correlation was observed between the area of blood flow and the day of assessment after 7 (IE: r = 0.43; *p* < 0.05) and 15 (IE: r = 0.52; *p* = 0.001; IN: r = 0.42; *p* = 0.001) days of transplantation. In conclusion, color Doppler ultrasonography can be used for real-time assessment of local blood perfusion in ovarian grafts, making it possible to identify alterations in the blood flow area in the period following a transplant procedure.

## Simple Summary

For this research, eight ovary pairs were surgically removed from goat donors, fragmented, and grafted in in-ear (IE) and in-neck (IN) areas for 7 or 15 days in goat recipients. On average, four ovarian pieces were transplanted in the IE and IN areas on both flanks (left: IE-7 and IN-7/right: IE-15 and IN-15). Color Doppler ultrasonography was used in this study to monitor the blood flow in the areas around the goat ovarian grafts. Ultrasound examinations were performed in all areas from day 0 to day 15. It was observed that the blood flow area was larger in the IE area compared to the IN area after 7 and 15 days of transplantation. The blood flow area was directly correlated with the day of evaluation in the IE area after 7 and 15 days and in the IN area after 15 days. Thus, it can be said that Doppler ultrasonography is a helpful tool to evaluate blood perfusion in ovarian grafts, allowing identification of changes in blood flow area.

## 1. Introduction

Transplant means the act of transferring organs or parts of tissues removed from a donor to a particular recipient using surgical techniques consistent with the transplant. Ovarian transplants are classified according to the recipient species: autotransplantation (a transplant to another part of the body in an individual), isotransplantation (between genetically identical animals), allotransplantation (between individuals of the same species), or xenotransplantation (transplanting between different species) [1]. Regarding the area, possible classifications include orthotopic, when the transplanted tissue or organ is reimplanted near the anatomical region of origin, and heterotopic, when the implant area is far from the original anatomical position [2].

In recent years, the use of heterotopic transplantation for ovarian tissue research has been increasing [3]. This technique has advantages over orthotopic implantation, as it presents less risk to the recipient and is less complex in terms of surgical and anesthetic invasiveness. This optimizes the procedure time, allowing multiple fragments to be implanted at different times, and allows easy monitoring of the implantation region [4,5]. Despite these advantages, opinions on the heterotopic ovarian implantation technique are divided, as it is limited to low oocyte development and requires the application of the in vitro fertilization technique to produce embryos [6,7,8].

Ovarian tissue transplantation has a single objective: maintenance of ovarian structure and physiology, which benefits multiple target patients in different situations. Ovarian transplant may be a viable alternative to resume endocrine function interrupted by the gonadotoxic action of radio- and chemotherapy in young women during cancer treatment [9,10]. Given that the primordial follicles of the ovary are located in the outer fibrous cortex, a simple graft procedure can be used to potentially restore ovarian function.

The advantages include a potentially large source of follicles and the rapidity with which the procedure can be performed. Employing ovarian tissue transplantation techniques associated with cryopreservation, more than 130 births have been reported [10].

Despite the encouraging results, some drawbacks remain. One of the most important disadvantages of ovarian tissue transplantation is related to ischemia, which can cause follicular loss [11]. Reperfusion plays a crucial role in follicle depletion in the first week after transplantation [7,12]. In the initial seven days after transplantation, oxygen and nutrients from the surrounding tissue diffuse toward the graft, rescuing it from ischemia and hypoxia. This process persists until the formation of new blood vessels, both inside the transplant and in the surrounding tissue, culminating in the completion of the revascularization process [13].

Evaluations of ovarian tissue after transplantation can be performed using different techniques, which allow the morphofunctional activity of the tissue to be monitored, thus providing valuable information about the performance of the procedure. These evaluations are usually performed using histology and immunohistochemistry techniques. Histology is a morphological assessment tool that allows microscopic observation of tissue architecture using specific stains. Immunohistochemistry uses morphological, molecular, and immunological principles to identify specific antigens through antigen–antibody interactions with high specificity, sensitivity, and accessibility [14]. However, these methods do not allow in vivo monitoring of transplanted tissues.

B-mode ultrasonography allows non-invasive real-time monitoring of ovarian structures in small ruminants that are unstimulated [15] or hormonally stimulated [16]. Regarding the transplantation of ovarian tissue in humans, two-dimensional ultrasonography was used to monitor follicular development [17], as well as to monitor embryonic development [18,19]. A valuable variation of ultrasonography that is still poorly explored in the context of ovarian transplants is the color Doppler, which provides real-time imaging of vascular dynamics by capturing the movement of erythrocytes around a transducer [20]. In this way, it captures information about the vascular architecture and the hemodynamic aspects of blood vessels examined in several organs, tissues, and structures of the reproductive system [20,21].

Three main modes are used during Doppler ultrasonography—spectral, power, and color Doppler—and each can be used to assess different blood parameters. In color Doppler mode, the Doppler shift can also be visualized as color signals superimposed over a conventional B-mode image. The Doppler effect in ultrasound is based on the fact that when ultrasound waves collide with moving structures, the frequencies of the reflected waves are different from the emitted ones, unlike when these waves collide with static surfaces, whose reflections are the same as the waves emitted by a transducer. The Doppler effect is proportional to the speed of movement of the reflecting surface [22].

Doppler ultrasonography allows the characterization and measurement of blood flow and can be used to indirectly make inferences regarding the functionality of different organs, including the ovaries. Several studies highlighted the importance of adequate blood flow for follicle development, ovulatory potential acquisition, and progesterone secretion by the corpus luteum. In sheep, for example, Doppler ultrasonography allowed researchers to infer that there is a maximum level of follicular blood flow that negatively affects the quality of an oocyte [23].

Nevertheless, studies carried out following ovarian transplantation in goats to verify the blood flow around grafts are very limited [24]. Thus, the aim of this study was to evaluate the effectiveness of color Doppler ultrasonography for examination of the blood flow areas in superficial graft areas after 7 or 15 days of heterotopic allotransplantation, comparing an in-ear subcutaneous area (IE) with an in-neck cervical intramuscular area (IN) in goats.

## 2. Materials and Methods

### 2.1. Ethical Aspects and Origin of Study Animals

The Ethics Committee for Animal Use of the INTA University Center—UNINTA approved all experimental procedures (protocol No.: 2022.08.01-P), and the goats were treated in accordance with the guidelines for the care of animals [25]. The experiment was conducted in the Veterinary Hospital of Large Animals in Sobral-CE, Brazil, at 3°40′58″ S, 40°21′4″ W. This study was performed with adult goats of the Savannah (n = 4) and Anglo-Nubian (n = 4) breeds as recipients and eight mixed-breed goats as ovarian donors. Before the study, the animals were dewormed with sodium closantel and vaccinated for rabies and clostridiosis. The females were raised under a semi-intensive production system. They had daily access to a Tifton (*Cynodon dactylon*) pasture in the morning and received hay of this grass in stalls. In addition, aiming to increase their body condition scores, their diet was supplemented with a commercial corn and soybean meal-based concentrate (18% crude protein). Water and mineral salts were furnished ad libitum. None of the animals had previously been utilized for ovarian stimulation.

### 2.2. Experimental Design

Sixteen goat ovaries were obtained from goats without defined breeds through surgical procedures. Before the ovariectomies, feed and water were withheld from the goats for at least 24 h and 12 h, respectively. Immediately before the ovariectomies, the abdominal areas anterior to the udders of the ovary donors were shaved and sprayed with a 2% iodine solution and 70% alcohol. Subsequently, an anesthetic protocol involving 1% atropine sulphate (0.044 mg/kg/SC, Sultropin^®^, Medinfar-Sorológico-Produtos e Equipamentos SA, Portugal) as a pre-anesthetic drug was applied. Ten minutes later, 2% xylazine hydrochloride (0.22 to 0.3 mg/kg/IM, Xilazin^®^ 2%, Madevet Comercial Agropecuária Ltd.a, Rio Grande do Sul, Brazil) was administered combined with 10% ketamine (7.5 mg/kg/IM, Cetamin^®^, Agener União Química Farmacêutica Nacional S/A, São Paulo, Brazil). Ten additional minutes later, 2% lidocaine hydrochloride (4.4 to 10.0 mg/kg, Lidovet^®^, Bravet Laboratory Ltd.a, Rio de Janeiro, Brazil) was applied via the high epidural route. Next, 6–8 mL of 2% lidocaine hydrochloride (Anestt^®^, LPS Agrofarma, São Paulo, São Paulo, Brazil) was applied SC on the incision line, which was on the alba line and measured 7 to 15 cm. The reproductive tract was then exposed, and the ovaries were removed. The suturing of several tissues was performed according to a simple standard protocol in three planes [26].

The ovaries were submitted to washes in alcohol, a sterile solution, and Minimal Essential Medium (MEM; Sigma Chemical Co., St. Louis, MO, USA) supplemented with antibiotics. Finally, the organs were transferred and preserved in MEM until fragmentation with a 2 mm punch to obtain cortical fragments [27] that were grafted. Then, on average, four fragments were transplanted per area, totaling 16 fragments per recipient animal, and they were grouped into different allotransplantation treatments: in the ear and in the neck for 7 (IE-7; IN-7) and 15 (IE-15; IN-15) days. The left and right grafts were removed after seven and fifteen days, respectively.

### 2.3. Allotransplantation

The female goats were deprived of food and water for 12 h and 8 h before the surgeries. The surgical procedure was based on [25]. Animal suffering was avoided by implementing the surgical procedure under anesthesia with 0.2 mg/kg/IM of xylazine hydrochloride (Xilazin^®^ 2%, Madevet Comercial Agropecuária Ltd.a, Rio Grande do Sul, Brazil) and 10 mg/kg/IM of ketamine hydrochloride (Ketamin^®^ 10%, Agener União Química Farmacêutica Nacional S/A, São Paulo, Brazil) associated with local administration of 2% clorhydrate lidocain (Anestt^®^, LPS Agrofarma, São Paulo, Brazil) at a dose of 2.5 mg/kg applied to the implant areas.

Small incisions were made in the skin at the graft sites (ear and neck). The fragments were then transplanted into the subcutaneous spaces of the ear and the splenic muscle of the neck for a total of 16 fragments per animal. The animals were intramuscularly injected with antibiotics and anti-inflammatory drugs. The same fasting and anesthesia protocol described above was used to harvest the tissues.

### 2.4. Assessment of Graft Revascularization Using Doppler Ultrasound

**Figure 1:**
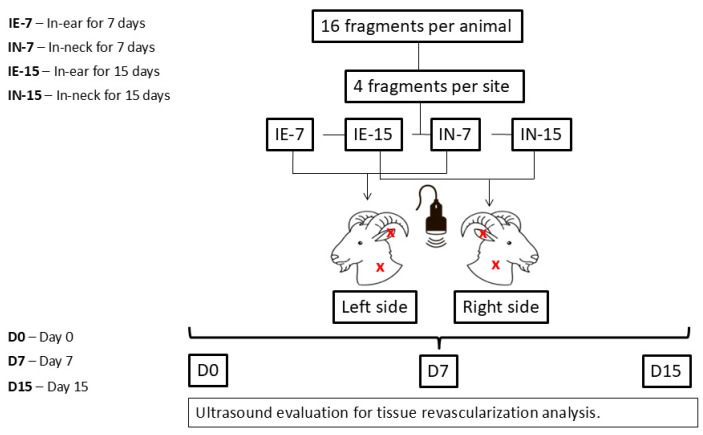
Color Doppler ultrasonography for examination of blood flow areas in superficial ovarian grafts. The red crosses represent the grafting points of the fragments on the goats.

All imaging examinations were performed by the same previously trained individual using a duplex portable color Doppler ultrasound device (M6VET, Mindray, Shenzhen, China) connected to a 5.0 to 10 MHz convex probe. The ultrasonographic evaluation was conducted with the goats standing, and after application of a water-soluble gel, the probe was positioned parallel to the surgical suture. To reduce the time required for the examinations, all ultrasonographic observations were recorded in video clips (≤10 s) and analyzed retrospectively. For all ultrasound examinations, the frequency settings (7.5 MHz), gain standards (20 dB), and color of the Doppler scale were kept constant throughout the study. Ultrasound examinations were performed in both areas (IE and IN) to monitor local blood flow, as shown in Figure 1. Color Doppler signals were assessed once a day (from day 0 pre-transplant to day 15 post-transplant) in the grafting areas until days seven and fifteen, when the left and right implants, respectively, were removed.

From the recorded videos, three different JPG images with maximum Doppler signals were obtained from the same region using the Gom Player program (Gretech Corporation, Seoul, Republic of Korea). The Image J program (National Institutes of Health, Millersville, PA, USA) was then used, after initial calibration (1 cm = 25 pixels), to measure the area (cm^2^) of the colored pixels in each image. The average of the areas obtained in the three images was then calculated.

### 2.5. Statistical Analysis

All analysis was performed by SigmaPlot version 11.0 (Systat Software, Inc., Richmond, CA, USA). To test for normality and homogeneity of variance, the data were subjected to the Shapiro–Wilk and Levene tests, respectively. A two-way ANOVA followed by Fisher’s LSD test was used to verify the effects of transplantation area and day on the area in pixels evaluated by ultrasound. The association between the variables post-transplant day and area in cm^2^ was analyzed using the Pearson correlation test. All results are reported as mean values ± the standard errors of the means (±SEM). Differences were statistically significant at the *p* < 0.05 level.

## 3. Results

Overall, the areas of blood flow around the transplanted ovarian fragments, as assessed by Doppler ultrasound, were statistically significant (*p* < 0.05) in the IE area in comparison to the IN area after 7 (IE: 4.70 ± 0.33^A^ vs. IN: 3.67 ± 0.33^B^) and 15 (IE: 5.27 ± 0.21 ^A^ vs. IN: 4.66 ± 0.22^B^) days of transplantation.

**Figure 2:**
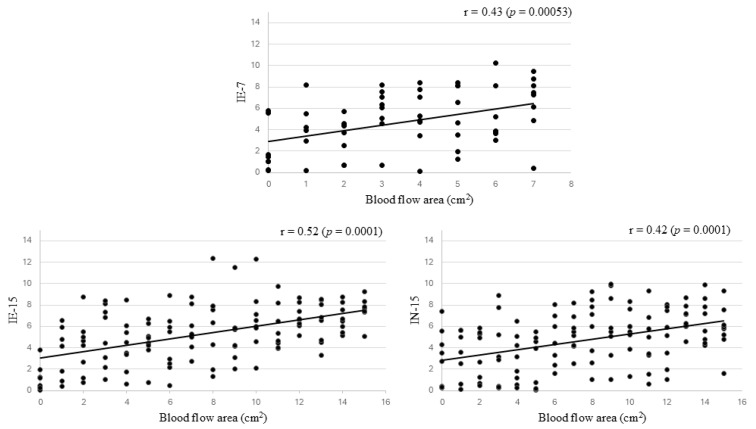
The relationship between the blood flow area (cm^2^) and the day of assessment after 7 and 15 days of ovarian transplantation in goats.

The daily ultrasound evaluation showed that the blood flow areas increased significantly in all evaluated regions when comparing day 0 (pre-transplant) with the last days of evaluation, 7 and 15 days after transplantation, as shown in Table 1. Therefore, this study reinforces the importance of vascular monitoring of transplanted tissue with the aid of Doppler ultrasound. A positive and significant correlation was observed between the area of blood flow and the day of assessment after 7 (IE: r = 0.43; *p* < 0.05) and 15 (IE: r = 0.52; *p* = 0.001; IN: r = 0.42; *p* = 0.001) days of transplantation, as shown in Figure 2.

## 4. Discussion

This work used color Doppler ultrasonography to examine local vascular perfusion in superficial graft areas after 7 or 15 days of heterotopic allotransplantation, comparing IE and IN areas in goats. The Doppler modality of ultrasound imaging allows for blood flow detection and quantification. In a previous review [28], it was reported that the application of Doppler ultrasonography in females of small ruminant species is significantly less widespread in comparison to large animals, mainly due to difficulties associated with transrectal and transcutaneous detection and visualization of internal reproductive organs.

Concerning ovary studies, early investigations performed during the estrous cycle reported substantial changes in ovarian blood flow in mares [27] and ewes [29]. Indeed, Doppler ultrasound has been used extensively in cattle to assess luteal and follicular blood perfusion. In a study conducted with cattle, greater vascular perfusion of preovulatory follicles was observed in follicles that resulted in cleaved versus non-cleaved oocytes during in vitro fertilization. Similarly, in stimulated goats, Doppler ultrasonography revealed that velocimetry parameters have positive associations with the follicular population and oocyte quality [30].

Regarding ovarian transplant experiments, investigations of angiogenesis are critical. In the past, immunohistochemical studies employing specific angiogenic markers were the main approach available to observe changes in angiogenesis [28]. Nowadays, accurate measurements of the blood flow in capillary networks draining reproductive organs can be achieved non-invasively in real time by color Doppler ultrasonography combined with computerized image analysis [31]. Doppler ultrasound has been shown to be a non-invasive and efficient technique for monitoring blood flow in ovarian graft areas in studies carried out by our team in horses [32] and goats [24]. In the latest study, it was observed that (i) blood flow was similar among intramuscular and subcutaneous areas of neck grafting and (ii) the amounts of blood flow were positively correlated with the percentages of normal follicles in the examined regions.

The use of the IE area for ovarian transplantation was previously successfully reported in mice [33]. After 3 weeks of grafting, there was a notable decrease in connective tissue density and an increase in ovarian blood vessels compared to observations made after 3 days. These authors observed a decrease in fibrosis density and an increase in ovarian vascularization after 3 weeks of grafting compared to 3 days. These researchers suggest that ear transplantation is advantageous because the ear is a more vascularized region capable of promoting a suitable environment for oocyte development. Recently, our team has shown that implantation in the subcutaneous region of the ear and intramuscular transplantation in the neck allow activation of the follicular pool observed in the tissue after 7 days of transplantation [34].

In our study, the increased blood flow observed in the grafting areas in all transplantation treatments probably promoted favorable environments for the ovarian tissue. Timely angiogenesis and effective perfusion of the microcirculation are essential for the survival and functional recovery of grafted ovarian tissue, thus resulting in higher success rates [35]. On the other hand, the occurrence of ischemic lesions after transplantation causes a large amount of follicular damage, which can seriously affect the restoration of ovarian function. In mares, lower follicular density was reported after ischemic events that occurred immediately after transplantation of frozen ovarian fragments [32].

## 5. Conclusions

In conclusion, color Doppler ultrasonography can be used for real-time assessment of local blood perfusion in ovarian grafts, making it possible to identify changes in blood flow area in the period following a transplant procedure. Thus, the color Doppler modality is a useful tool to predict which anatomical transplantation area will be most favorable to follicular development.

## Figures and Tables

**Table 1:** Means ± SEM of areas (cm^2^) of blood flow assessed by Doppler ultrasound from day 0 to day 15 after transplantation in in-ear (IE-7 and IE-15) and in-neck (IN-7 and IN-15) areas in goats.

	Treatment			
Day	IE-7	IN-7	IE-15	IN-15
0	2.11 ± 0.91^A^	2.53 ± 091^A^	1.16 ± 0.85^A^	3.05 ± 0.85^ABC^
1	4.15 ± 1.05^ABC^	4.42 ± 0.97^AB^	3.58 ± 0.85^B^	3.31 ± 0.91^ABCD^
2	3.36 ± 0.91^AB^	3.40 ± 0.91^AB^	4.07 ± 0.85^BC^	3.32 ± 0.85^ABCD^
3	5.67 ± 0.91^BC^	3.78 ± 0.91^AB^	5.17 ± 0.85^BCDEFG^	3.61 ± 0.85^ABCDE^
4	5.20 ± 0.91^BCa^	2.32 ± 0.91^Ab^	4.22 ± 0.85^BCD^	2.84 ± 0.85^AB^
5	5.32 ± 0.91^BC^	3.28 ± 0.91^AB^	4.51 ± 0.85^BCDEF^	2.50 ± 0.85^A^
6	5.21 ± 0.91^BC^	4.44 ± 0.97^AB^	4.36 ± 0.85^BCDE^	4.69 ± 0.91^ABCDEF^
7	6.55 ± 0.91^C^	5.22 ± 0.91^B^	5.65 ± 0.85^BCDEFGH^	5.29 ± 0.85^CDEF^
8	-	-	6.19 ± 0.85^CDEFGH^	5.76 ± 0.85^EF^
9	-	-	5.30 ± 0.85^BCDEFGH^	6.14 ± 0.85^F^
10	-	-	6.60 ± 0.85^EFGH^	5.47 ± 0.85^DEF^
11	-	-	5.85 ± 0.85^BCDEFGH^	4.47 ± 0.85^ABCDEF^
12	-	-	6.89 ± 0.85^GH^	5.11 ± 0.85^BCDEF^
13	-	-	6.37 ± 0.85^DEFGH^	6.71 ± 0.85^F^
14	-	-	6.78 ± 0.85^FGH^	6.56 ± 0.85^F^
15	-	-	7.57 ± 0.91^H^	5.78 ± 0.85^EF^

^A, B, C, D, E, F, G^ and ^H^ Within a column, values without a common superscript are different (*p* < 0.05). ^a, b^ Within a row, values without a common superscript are different (*p* < 0.05).

## Data Availability

The data presented in this study are available within the article’s figures and table. No new data were created or analyzed in this study.
